# Valuing health states: is the MACBETH approach useful for valuing EQ-5D-3L health states?

**DOI:** 10.1186/s12955-018-1056-y

**Published:** 2018-12-18

**Authors:** Mónica Duarte Oliveira, Andreia Agostinho, Lara Ferreira, Paulo Nicola, Carlos Bana e Costa

**Affiliations:** 1 Centre for Management Studies of Instituto Superior Técnico - CEG-IST, Universidade de Lisboa, Lisbon, Portugal; 2 University of the Algarve – ESGHT, Faro, Portugal; 3 Centre for Health Studies & Research – CEISUC, University of Coimbra, Coimbra, Portugal; 4 Epidemiology Unit, Faculty of Medicine, Institute of Preventive Medicine, Universidade de Lisboa, Lisbon, Portugal

**Keywords:** QALY, Preference-based instruments, Health states valuation, MACBETH, TTO, I120, I190

## Abstract

**Background:**

Quality Adjusted Life Years (QALYs) are a key outcome measure widely used within health technology assessment and health service research studies. QALYs combine quantity and quality of life, with quality of life calculations relying on the value of distinct health states. Such health states’ values capture the preferences of a population and have been typically built through numerical elicitation methods. Evidence points to these value scores being influenced by methods in use and individuals reporting cognitive difficulties in eliciting their preferences. Evidence from other areas has further suggested that individuals may prefer using distinct elicitation techniques and that this preference can be influenced by their numeracy. In this study we explore the use of the MACBETH (Measuring Attractiveness by a Categorical Based Evaluation Technique) non-numerical preference elicitation approach for health states’ evaluation.

**Methods:**

A new protocol for preference elicitation based on MACBETH (only requiring qualitative judgments) was developed and tested within a web survey format. A sample of the Portuguese general population (n=243) valued 25 EQ-5D-3L health states with the MACBETH protocol and with a variant of the time trade-off (TTO) protocol, for comparison purposes and for understanding respondents’ preference for distinct protocols and differences in inconsistent evaluations. Respondents answered to a short numeracy test, and basic socio-economic information collected.

**Results:**

Results show that the mean values derived from MACBETH and the TTO variant are strongly correlated; however, there are substantial differences for several health states’ values. Large and similar numbers of logical inconsistencies were found in respondents’ answers with both methods. Participants with higher levels of numeracy according to the test preferred expressing value judgments with MACBETH, while participants with lower levels were mostly indifferent to both methods. Higher correlations between MACBETH and TTO variant evaluations were observed for individuals with higher numeracy.

**Conclusion:**

Results suggest that it is worth researching the use of non-numerical preference elicitation methods. Numeracy tests more appropriate for preference elicitation when no explicit considerations of uncertainty are made need to be explored and used. Further behavioural research is needed to fully understand the potential for using these methods in distinct settings (e.g. in different evaluation contexts and in face-to-face and non-face-to-face environments), as well as to explore the effect of literacy on assessments and on respondents’ preferences.

## Introduction

In a context of limited resources and of an increasing demand for health care, much attention is being paid to how to best spend available resources, with health technology assessment being a growing field with the responsibility for developing tools and knowledge to inform resource allocation [1]. Within health technology assessment, conventional economic evaluation techniques, such as cost-benefit analysis, cost-effectiveness analysis and cost-utility analysis, have been widely used to compare two or more alternative technologies in terms of their costs and health benefits [2]. Among these techniques, the use of cost-utility analysis has been growing since it captures how extra resources invested in one health technology relate to extra health gains, with these being valued by the single summary known as the Quality Adjusted Life Years (QALYs) utility measure. The use of QALYs has been of particular interest since it enables comparisons across a wide range of health technologies and interventions (enabling allocative efficiency analyses), and it is based on individuals or patients’ preferences across distinct health-states. In fact, QALYs are a health outcome measure used not only by several health technology assessment bodies in health technology evaluations [3] but also in several clinical and patient decision-making contexts [4].

Behind the QALYs health outcome measure is the calculation of the length and quality of life associated with a patient using one health care technology, e.g., the patient’s life expectancy multiplied by the quality of life in those remaining years. Changes in quantity of life are expressed in terms of survival or life expectancy and measured in years, while the quality of life adjustment for each year lived is based on a set of preference values or weights called utilities that are calculated for different health states, capturing the health states value or desirability [5, 6]. These QALY utilities are measured on an interval scale, where 1 refers to full health and 0 refers to death, and in which it is possible to have severe health states worse than death and thus assuming negative utilities [2, 7].

Three conventional methods have been commonly used for directly eliciting preferences for health states, the visual analogue scale, the Time Trade-Off (TTO) and the standard gamble. These methods entail numerical protocols for questioning – asking individuals to provide direct or indirect numerical assessments of health states – and their use has been shown to lead to different results, there being no consensus regarding the most adequate method and with the literature acknowledging distinct pros and cons associated with each method [8, 9]. The visual analogue scale has been recognized as the simplest method, with respondents rating health states at points on a (visual) line that has two reference points, usually a most preferred and a least preferred health state, and locating on the line those health states so that they capture how much better and/or worse those health states are believed to be in relation to the two references [10]. Nevertheless, the visual analogue scale is not a choice-based technique and has been recalled to have the weakest theoretical foundations [6, 11]. Given its simplicity, it has often been used as a “warm-up” exercise before other methods are applied. The standard gamble makes respondents choose between decision options which involve uncertain outcomes [12] – for instance, it causes respondents to express indifference between the certainty of a health condition and the risk of immediate death or of perfect health – and has been recognized as the classical method of measuring cardinal preferences because it is directly based on the axioms of utility theory [2]. Similar to the standard gamble, the TTO has also been called a choice-based technique in which respondents consider the number of life years they would be willing to sacrifice to avoid a certain poorer health state [2, 11, 13]. Although the TTO and the standard gamble are the two most widely used methods to measure patients’ values and utilities (respectively) across health states, some drawbacks associated with its use have been pointed out [7, 14, 15], namely, responses are likely to be influenced by factors such as risk behaviour of the respondents, time preference or aversion to loss; protocols are complex and demand a high cognitive effort; and they make use of different procedures to evaluate states better or worse than death.

To build an instrument that can be extensively used to evaluate a comprehensive set of health states, descriptive systems have been developed so that the health status of the individual is classified on a common ground and an algorithm for assigning value scores to each health state is described by the system [16] (with those value scores being based on preferences of the general public). Examples are the Quality of Well-Being, the Health Utilities Index, EuroQol-5D (EQ-5D-3L) and the Short-Form 6D (SF-6D) [6]. These instruments differ in terms of the health dimensions included, the number and description of levels defined for each dimension, the population on which the preferences are based, and in terms of the valuation method with which they are combined. For instance, the TTO was used to value the EQ-5D-3L system while the standard gamble was used to value the Health Utilities Index and the SF-6D [6]. These instruments have become widely used in economic evaluation, and have thus enhanced the use of QALYs.

In addition to the numerical preference elicitation protocols just described (visual analogue scale, TTO and standard gamble), ongoing research has been developing alternative measurement techniques for the elicitation of health-state values. Examples of these techniques are ordinal methods such as discrete choice experiments and ranking exercises that may offer advantages such as ease of comprehension and administration and a reduced cognitive burden which are particularly important in settings in which evaluators have limited educational attainment and low numeracy [15]. For instance, previous studies have focused on the use of ranking exercises to estimate value sets for the EQ-5D-3L [17], the Health Utilities Index [18] and the SF-6D [18, 19]. Recently, an international collaborative research group, from the EuroQol, investigated the potential of discrete choice approaches [20–22]. The new protocol developed by this EuroQol group to value EQ-5D-5L health states defines the use of both TTO and discrete choice methods [23] and valuation studies have been published using a model that combines both types of data [24] or that uses discrete choice experiments that qualify as non-numerical methods [22] asking for ordinal preference information. Discrete choice experiments have also been used to value the SF-6D [25] and in other studies where preferences are elicited (e.g. [26]).

Regarding the use of different elicitation protocols, although distinct techniques – ranging from numerical to non-numerical – can be deemed as theoretically equivalent for constructing interval value scales, such protocols may be perceived and experienced in distinct ways by evaluators [27]. In fact, distinct techniques may be seen as not being psychologically equivalent, as experimentally inferred in [27] by observing that evaluators’ inclination toward one technique is linked to their numeracy [28] and fluency [27]. Previous behavioural studies have actually shown that numeracy affects how people make decisions under uncertainty contexts [29, 30] and may influence values obtained through conventional preference elicitation techniques and the preferred mode of expressing value judgments, in numbers or words [27, 28, 31]. A study in the context of multi-criteria decision analysis has also suggested (although not experimentally assessing) that the preference for verbal versus numerical aiding techniques is affected by the expertise of those facilitating model building, as well as by the participants’ education [32], and decision analysis practitioners have reported that numerical and non-numerical techniques are not equally accepted by users and can be rejected by some and endorsed by others [33]. Health-state evaluation literature has not explored the use of non-numerical protocols for questioning, and there is the possibility for producing behavioural studies to understand respondents’ preferences for numerical and non-numerical protocols.

Aiming to fill this gap, this article is a pilot study about an innovative and unconventional approach to evaluating health states, based on the use of the MACBETH (qualitative) questioning mode for valuing EQ-5D-3L health states; and the study develops behavioural research testing for individuals’ preferences for numerical and non-numerical questioning protocols, exploring whether numeracy relates to their preferences, and whether numeracy influences health states’ values and consistency in assessments. MACBETH stands for *Measuring Attractiveness by a Categorical Based Evaluation Technique* and is a non-numerical preference elicitation approach [34] and a difference-value measurement technique with sound theoretical foundations (originally introduced in 1994 [35] and last updated in 2012 [34], its theoretical foundations are described in [36]). MACBETH has been widely used to support decision-making in multiple public and private contexts and sectors, including the health sector for the prioritization and selection of health-care programmes [37], the evaluation of occupational health and safety risks [38], evaluating patients’ preferences for pharmaceuticals [39], evaluating technologies in the context of regulatory health technology assessment [40–42], the evaluation of cardiovascular treatments for paediatric patients in terms of equipment [43], diagnosing Alzheimer’s disease [44], informing maintenance policies in health-care organizations [45, 46], and for building a population health index [47]. At the core of MACBETH is the measurement of the relative value of options through an intuitive questioning protocol based upon pairwise comparisons of differences in preference (attractiveness or desirability) between options in the following semantic categories: no difference, very weak, weak, moderate, strong, very strong or extreme difference [34]. The M-MACBETH decision support system [48] assists in assessing the qualitative judgments, testing their consistency, and converting them into numerical value scores (as detailed in section 3.1).

In this article MACBETH is used to evaluate health states, as defined by the EQ-5D-3L classification system: the EQ-5D-3L is an instrument that categorises respondents’ health statuses in five dimensions (mobility, self-care, usual activities, pain/discomfort and anxiety/depression), with each dimension entailing three severity levels (no problems, some problems and severe problems), which in total define 243 health states [49]. An extra “death” health state is usually added to the classification. EQ-5D-3L is commonly coded as a combination and sequence of those five dimensions and, within each dimension, the “no problems” level is coded as 1, the “some problems” level as 2, and the “severe problems” level as 3: for instance, the 12111 health state means that the individual has no mobility problems, has moderate self-care problems, and has no problems regarding usual activities, pain/discomfort and anxiety/depression. As EQ-5D-3L health states correspond to a nominal level of measurement (since they cannot be ordered and have no intrinsic quantitative value score), MACBETH is a path towards measuring health states into a cardinal value scale [35], and assign index values to individual health states.

In this article, we specifically explore the use of the MACBETH questioning mode for valuing EQ-5D-3L health states in a web survey format. This survey is also designed to investigate the impact of individuals’ numeracy on the choice of methods and to evaluate health states, namely, to address the following hypotheses:

- Does numeracy affect individuals’ preferences between numerical and non-numerical preference elicitation techniques?
- Does a population’s numeracy influence values obtained through numerical and non-numerical techniques?
- Does numeracy increase consistency in health-state evaluations obtained through different numerical and non-numerical techniques?

While a hypothesis similar to a) has been searched in a different context in [27], the studies [28, 31] have explored hypotheses similar to b), and [31] explored a hypothesis similar to c).

This study contributes to literature in several ways: it explores a non-numerical technique which has the potential to overcome perceived limitations of conventional valuation techniques, i.e. regarding cognitive effort [12]; and as a behavioural study it tests the extent to which respondents’ prefer numerical and non-numerical techniques, whether numerical and non-numerical protocols influence health-state evaluations and consistency, and whether numeracy influences results. Results can potentially be relevant to health research literature as the EQ-5D-3L is a widely used instrument [49] with applications in multiple health areas (e.g., clinical studies, population health surveys, economic evaluation of health care [2]) and is recommended by multiple institutions as a health outcomes measure [50]. The values produced by health preference elicitation studies are critical inputs for measuring health technologies’ benefits using mainstream cost-utility analysis approaches, with variations in inputs raising cost-utility robustness issues.

## Methodological framework

To address the above hypotheses we designed a new questioning protocol, based on the MACBETH non-numerical elicitation approach, to valuing health states in a non-face-to-face (web) setting, together with the implementation of a variant of the TTO protocol for comparison purposes. The web survey was set to collect data about the preferred mode of expressing value judgments and to carry out a numeracy test. Procedures were defined to derive health-state evaluations from the MACBETH and TTO variant protocols and to identify exclusion criteria, as well as to analyse the web survey results.

### New elicitation protocol based on MACBETH

**Fig. 1:**
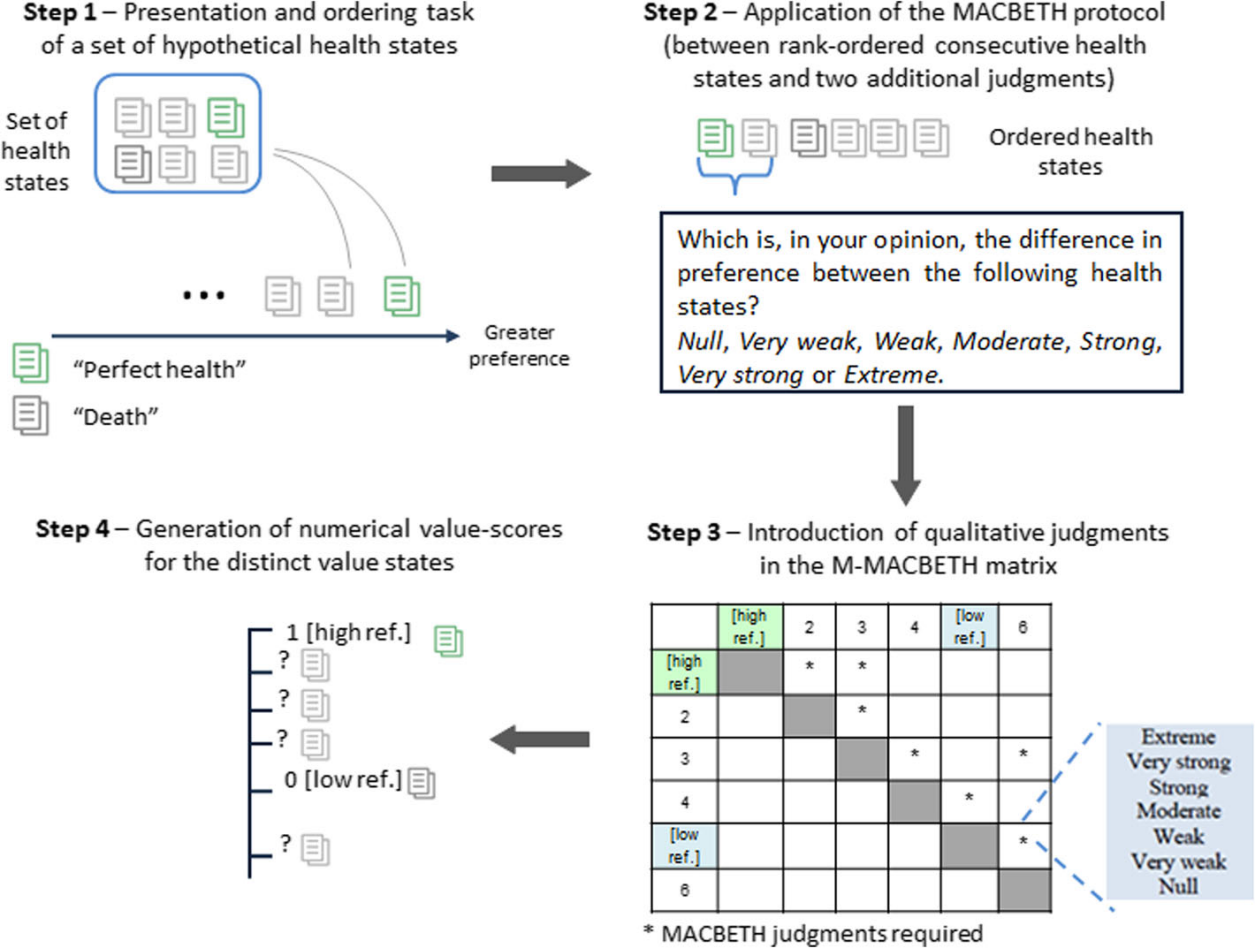
Schematic representation of the steps followed to implement the MACBETH protocol in a web survey environment [health states are generically portrayed as 1 (=[high ref.]), 2, 3, 4, 5 (=[low ref.]) and 6)]

The rationale for using the MACBETH approach in the valuation of health states is its intuitive questioning protocol that only requires qualitative judgments about differences in preference between pairs of health states for building numerical scores. Applying the protocol to the health states evaluation context, the following question can be used: “given two health states *x* and *y*, with *x* better than *y*, which is the difference in preference between *x* and *y*? In your answer make use of the following categories: *null*, *very weak*, *weak*, *moderate*, *strong*, *very strong* and *extreme*”. The elicited judgments are introduced in the M-MACBETH decision support system [48] filling a matrix of judgments like the one in the bottom left of Fig. 1 which depicts, for illustrative purposes, the evaluation of six – from *1* to *6* – health states [48]. In this matrix it can be seen that health states are rank-ordered from more to less attractive *1 (=[high ref.])*, *2*, *3*, *4, 5 (=[low ref.])* and *6*, and that some of these health states are pairwise compared using MACBETH qualitative judgments (from *null* to *extreme*).

Generically, for a set of *n* health states that are ordered in the MACBETH matrix of judgments by decreasing order of preference, it is not necessary to perform all of the n (n-1)/2 pairwise comparisons and populate the upper triangular part of the matrix completely. The minimal number of judgments required is n-1, e.g., assessing differences in preferences between one state and each of the others, or assessing differences between all consecutive rank-ordered states. However, it is recommended that additional judgments be requested, so that consistency checks are performed and a higher precision in numerical assessments can be obtained [34].

Once MACBETH qualitative (consistent) judgments are elicited, the M-MACBETH decision support system assists in analysing which numerical scales are compatible with a set of qualitative judgments of differences in preference. Regarding the numerical scale, the higher the number of qualitative judgments asked of respondents, the higher the consistency of their numerical representation [51], although asking for a larger number of qualitative judgments can be time-demanding and translate into respondents’ tiredness. Typically (and ideally), when qualitative judgments are elicited in a face-to-face environment, the evaluator is then asked to analyse, eventually adjust and validate the numerical scale proposed by MACBETH. In cases where each qualitative judgment is introduced in the M-MACBETH matrix, M-MACBETH assists in testing consistency with other judgments, and when inconsistency is detected, it suggests ways to resolve inconsistencies [48]. Once a consistent set of judgments is obtained, M-MACBETH uses a mathematical programming algorithm to derive a numerical scale on the set of health states that is compatible with all the qualitative judgments, which is the starting point for the participant(s) validating numerical scores and eventually adjusting one or more health states to set a cardinal interval scale. Such conventional procedures need to be adapted when the MACBETH protocol is used in a web survey environment.

In this study, the MACBETH protocol for valuing health states is set to follow four steps, according to the schematic representation of Fig. 1. Implementing this protocol (described in detail in the next section), the first two steps relate to the web survey in which the evaluator participates, and in the remaining steps, the M-MACBETH decision support system is used to analyse evaluators’ answers. In particular, the first step consists of asking an evaluator to order a set of health states. Following health states literature, an interval scale is adopted, and thus the states “perfect health” and “immediate death” should be included in that set, and they are then used as “upper” and “lower” references with assigned values of 100 (or alternatively 1) and 0, respectively, in the calculation of health-states values. The second step consists of asking an evaluator for his/her qualitative judgments of differences in preference between various health states. In this study, we designed a process in which the evaluator is asked to compare consecutive rank-ordered health states (including the two references) as well as two additional judgments to check for logical consistency in evaluations. In the third step, the information provided by each evaluator is introduced in a MACBETH matrix of judgments, while in the last step, the numerical value scale depicting health-states evaluations for each respondent is obtained from the M-MACBETH decision support system (for consistent judgments). One should note that in this study the evaluator provides qualitative judgments and is not asked to adjust and validate the numerical scale, with results being based upon the hypothesis of cardinality of the MACBETH scale. In fact, under this hypothesis, the M-MACBETH decision support system is used to transform elicited MACBETH qualitative judgments into a numerical scale that is taken as tacitly accepted by the evaluator (see details of how that scale is calculated in [34]). This assumption is reasonable because in many practical exercises evaluators directly accept the numerical scale proposed by the M-MACBETH decision support system.

### Web survey

We have designed and implemented a web survey using the Qualtrics platform [52, 53]. The choice for an online survey format was motivated by its easy and quick distribution and by the numerous logistical challenges and resource limitations associated with the use of face to face methods. Given an increasing and widespread use of internet access, it is expected that such participatory formats will be increasingly explored in future valuation studies [54, 55].

The purpose of this survey was to implement the non-numerical MACBETH protocol and collect the qualitative judgments on EQ-5D health states, collect data about the evaluators’ preferences for providing numerical or non-numerical judgments, as well as to assess the evaluators’ level of numeracy. This later assessment enabled analysing the extent to which evaluations and preferences are influenced by numeracy and whether numeracy influences evaluations. For comparison purposes, we have also collected numerical judgments on the same EQ-5D health states obtained by applying a TTO questioning mode in a simplified format, as implemented in [56, 57], which we call TTO variant in this article.

**A. Selection of health states**

As mentioned earlier, the EQ-5D-3L descriptive system [6] consists of five dimensions (mobility, self-care, usual activities, pain/discomfort and anxiety/depression) with three possible levels each (level 1 – no problems; level 2 – some problems; level 3 – extreme problems), thus amounting to 243 (3^5^) possible health states plus the death health state. Health-states descriptions are constructed by taking one level in each dimension, e.g., 11111 represents the perfect health state and 33333 the combination of extreme problems in all dimensions [58].

Whenever appropriate, we have adopted the same experimental choices of the study that estimates the EQ-5D-3L value set using TTO for Portugal [56] – this option enables crossing our results with health-states scores from that study. Following that study, a sub-set of 24 hypothetical health states was chosen for valuation plus the health states 11111, 33333 and “immediate death”; and since previous studies have shown that respondents are not capable of valuing more than approximately 13 health states within the same exercise, the health states were divided into four equally sized groups according to their severity [57]. Each respondent was randomly assigned to one of those groups, with each group assessing distinct sets of health states as depicted in Table 1.

**Table 1:** EQ-5D-3L health states set assignments (groups from [56])

Group 1 health states	Group 2 health states	Group 3 health states	Group 4 health states
13311	12111	11113	21111
22222	11131	32313	23232
11112	32211	11211	11121
11133	21323	22121	11312
32223	22233	13332	33323
33321	23313	33232	22122
33333	33333	33333	33333
Immediate death	Immediate death	Immediate death	Immediate death

**B. Web survey structure and elicitation tasks**

Each respondent was asked to: 1) describe his/her own health according to the EQ-5D-3L descriptive system; 2) order and value the set of hypothetical health states using the MACBETH protocol and a variant of the TTO (tasks presented in random order); 3) complete a short numeracy test composed of three validated questions from the work of Woloshin and colleagues [28]; 4) state his/her preferred way of expressing value judgments (non-numerical with MACBETH, numerical with the TTO variant, or indifferent); and 5) report their socio-demographic characteristics.

Woloshin et al. numeracy questions [28], which have been used in several health-evaluation studies, were adopted: question 1 assessed familiarity with probability by asking about the number of heads in 1000 coin flips (answers between 470 and 530 were taken as correct answers (95% confidence interval)); question 2 asked respondents to convert 1% (a percentage) into a proportion (10 in 1000 as correct answer); and question 3 asked the respondent to convert the 1 in 1000 proportion into a percentage (0.1% as correct answer).

**Fig. 2:**
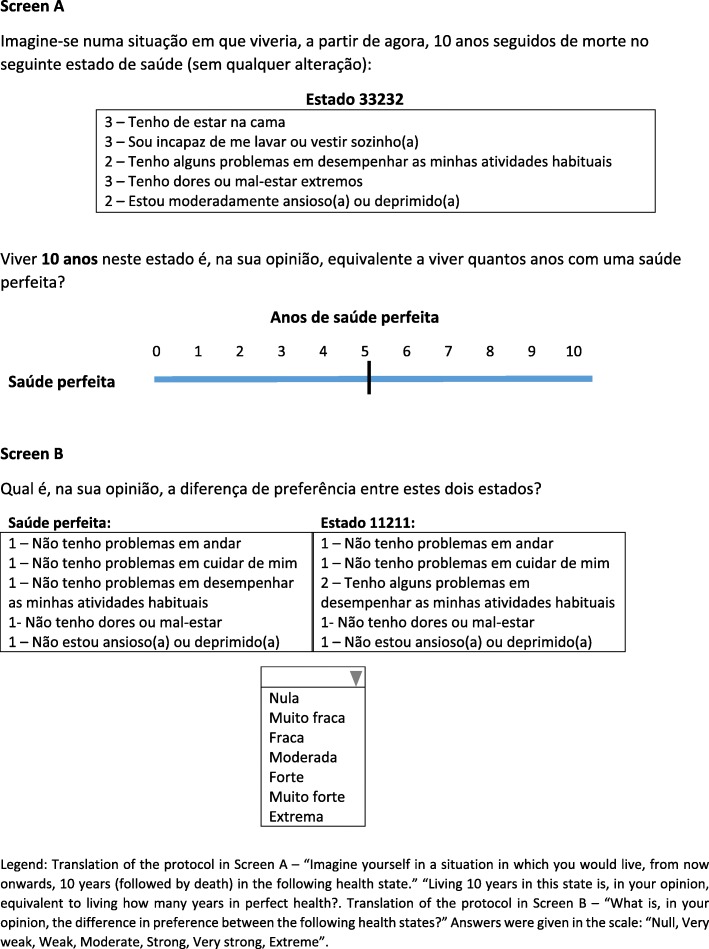
Web survey screens, with Screen A – TTO task for a health state better than dead, and Screen B – MACBETH task. The survey and all the tasks required are in Portuguese. [Legend: Translation of the protocol in Screen A – “Imagine yourself in a situation in which you would live, from now onwards, 10 years (followed by death) in the following health state.” Living 10 years in this state is, in your opinion, equivalent to living how many years in perfect health?. Translation of the protocol in Screen B – “What is, in your opinion, the difference in preference between the following health states?” Answers given in the scale: “Null, Very weak, Weak, Moderate, Strong, Very strong, Extreme”]

To value different hypothetical health states, each respondent was told that he/she had to imagine themselves in each state for a period that would last 10 years, after which he/she would die. A task example of the MACBETH protocol and of the TTO variant is shown in the prototype displayed in Fig. 2. The survey was administered in Portuguese.

With regard to the MACBETH protocol, following the two tasks described in Fig. 1, respondents first participated in the rank ordering exercise of all health states (including death) and were asked to select the best state, which then disappears from the screen, and to continue selecting health states until a full ordering is established; next, respondents were asked for pairwise qualitative judgments in screens such as the one depicted in Fig. 2 – Screen B. Specifically, the following protocol was applied “What is, in your opinion, the difference in preference between the following health states?” and answers were given in the semantic scale *Null*, *Very weak*, *Weak*, *Moderate*, *Strong*, *Very strong*, *Extreme*. Respondents were asked for 9 non-numerical judgments: for the consecutively ranked health states and for two extra pairwise comparisons.

Given the need to apply the TTO in a web survey in which there are time limits, we adopted a simplified TTO protocol from [56, 57]. This simplified TTO protocol that is a variant of the conventional TTO, consisted of two tasks. First each respondent was asked to indicate whether his/her health state was better or worse than death, with the following question being used: “Imagine yourself in a situation in which you would live, from now onwards, 10 years (followed by death) in the following health state.” “Living 10 years in this state is, in your opinion, equivalent to living how many years in perfect health?” (Screen A in Fig. 2). Following that response, respondents were asked to value states better than death and worse than death, as in [56]. Specifically, the process has been simplified through the direct appearance of a horizontal scale, limited by the 0 and 10, representing the number of years in full health (state better than death) or the number of years in the target health state (state worse than death). Each respondent was asked then to directly indicate the indifference point between alternatives, visualising a screen such as the one shown in Fig. 2 – Screen A. As discussed later, this simplification bears a conceptual resemblance to a visual analogue scale.

**C. Distribution strategy and target population**

The target population for the study consisted of the Portuguese general population, aged 18 and over, in which the proposed protocol was tested. The following strategy was followed: 54 individuals were recruited to participate in this study through an email invitation which contained a link for the survey; individuals were asked to forward the invitation to their contacts, with a non-probability sampling strategy being adopted, specifically a snowball (or networks) sampling [59]. The 54 individuals were defined as a diverse sample (in terms of age, gender and professional backgrounds) of personal contacts from the authors, and the survey was available from March to May 2015, a period that enabled obtaining a sufficient number of answers to perform the proposed analyses.

### Valuation procedure

Once data from the web survey was collected, numerical scores for the different health states were calculated. Regarding the TTO variant, perfect health and death were given the 1 and 0 values, respectively. Then, and following common practice in TTO studies, the scores *h* for health states better than death were calculated using the formula *h* = *t*/10, and for states worse than death, the formula *h* = (−10 + *t*)/*t* was adopted (in both formulas *t* represents the indifference point) [57].

Regarding the MACBETH questioning mode, qualitative judgments from each respondent were inserted in the M-MACBETH decision-support system, and if judgments were consistent, a numerical scale was produced (as described in Fig. 1, Steps 3 and 4). The numerical (interval) scale used as references “perfect health” and “death” with the 1 and 0 scores, respectively. In order to enable a comparison between MACBETH and TTO scores, as well as with health evaluations carried out in other studies, a monotonic transformation was applied to the MACBETH scores for states worse than death assuming negative values, thereby assuming a -1 lower bound and using the transformation *h*^′^ = *h*/(1 − *h*), as in [56]. One should, however, note that this adjustment is not commonly used in the application of the MACBETH approach – most commonly, by following psychometric theory and by considering the properties of an interval scale [60], an interval scale is anchored in two points and there are no lower bounds for numerical values below 0.

### Exclusion criteria

According to the literature [51, 56], respondents often provide inconsistent judgments, partly due to a lack of understanding or misinterpretation of questionings. The following procedures to deal with inconsistent answers were adopted: concerning answers to the numerical (TTO variant) protocol, respondents were excluded if 1) all states were valued worse than death; 2) all states were given the same value; 3) for states ordered better or worse than death, a score equal to zero was given; or 4) for states worse than death, a score equal to zero was given. Additionally, exclusion criteria based on “logical inconsistency” and “serious logical inconsistency”, previously defined in other studies [56], were adopted. That is, a “logical inconsistency” occurs at a respondent level if, among two pairs of health states, one health state is better than the other one at least in one dimension and not worse in any other dimension, and the valuation of the former state is worse than the valuation of the latter health state (with dominance principles being applied [61]); and a “serious logical inconsistency” occurs if the difference in valuation is greater or equal to 0.5.

Concerning answers to the MACBETH protocol, respondents were excluded in the following cases: 1) logical inconsistency resulting from the rank order exercise; 2) all states were valued worse than death; and 3) an inconsistent MACBETH matrix of qualitative judgments was obtained.

When exclusions were found for both protocols and for the same individual, the total questionnaire was excluded. In the other cases, responses to one protocol were considered for analysis. Note that the consistency requirements associated with the use of MACBETH are more demanding, and comparison of results should take this into account (more on this in the Discussion section).

To enable analysing a possible influence of order in the proportion of incomplete questionnaires and on exclusions, the order of presentation of the MACBETH and the TTO tasks in the web survey was randomized at the individual level.

### Data analysis

Data analyses included:

- the interpretation of descriptive statistics to characterise the sample according to its socio-economic characteristics, current health status, numeracy and preference for distinct questioning protocols;
- a comparison of health state value scores for the population sample obtained with numerical and non-numerical elicitation protocols, as well as with the results of the Portuguese EQ-5D-3L valuation study where the regular TTO was used [56]; correlation coefficients (Pearson and Spearman) were also included to compare value scales obtained with the two protocols;
- an analysis of inconsistencies in respondents’ answers when each protocol is used and in the numerical and non-numerical judgments provided by respondents. This included testing for each respondent whether the numerical TTO scale was compatible with qualitative MACBETH judgments using the M-MACBETH software;
- a study of the influence of numeracy on preferences, on health-state valuations and on the exclusion of answers. Comparisons of sociodemographic characteristics and current health status of the subgroups were made using parametric tests (t tests and ANOVA) and non-parametric tests (*χ*^2^ tests, fisher exact test and Mann-Whitney test). A multinomial logistic regression was applied to study the influence of numeracy in the preferences regarding the ways of expressing value judgments (numerical with TTO variant, non-numerical with MACBETH, or indifferent).

All statistical analyses were performed using the R software (v.3.1.3) [54, 62] and a 5% significance level was considered (*p* < 0.05).

## Results

### Obtaining a valid sample

**Fig. 3:**
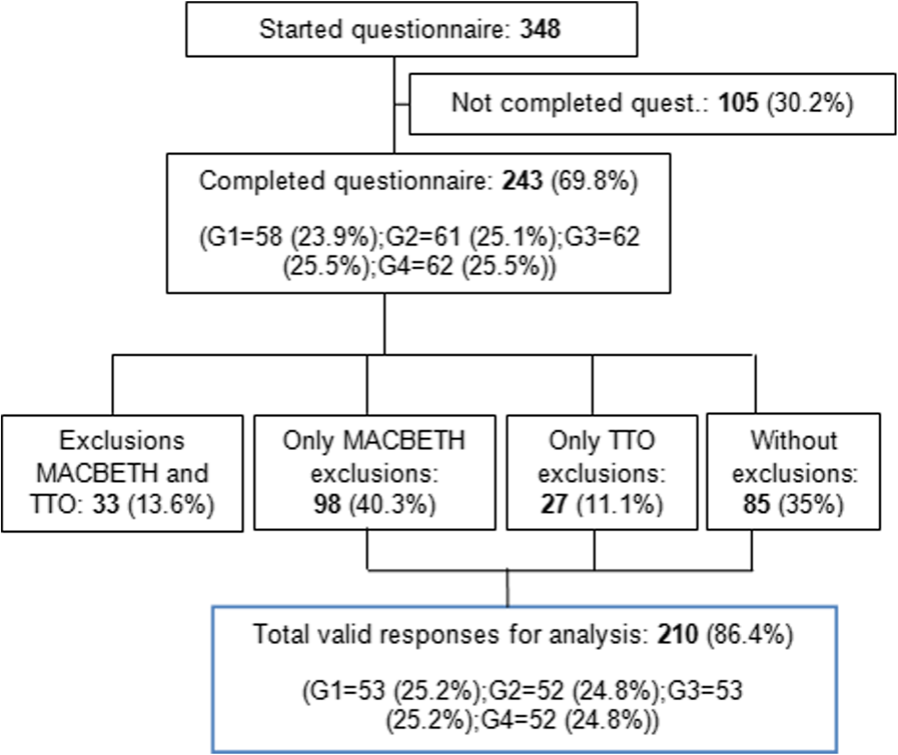
Flow diagram of the process of obtaining a valid sample population for analysis (G1, G2, G3, G4 – Set of responses obtained for each one of the four sets of health states depicted in Table 1)

Figure 3 shows a flow diagram describing the process of obtaining a valid sample population for analysis. After an initial email was sent to 54 individuals, a total of 348 individuals initiated the survey and explicitly accepted participation in our study. From these, only 243 completed the survey (completion rate of approximately 70%). Since this a pilot study, this number was deemed as sufficient to perform the proposed analyses and to test the proposed protocol – in fact, its size is similar to the ones used in other pilot studies proposing variants to existing protocols (e.g., [63–65]).

Given that the order of presentation of the MACBETH and the TTO tasks was randomized at the individual level, it was considered of interest to analyse a possible influence of order in the proportion of incomplete questionnaires and on exclusions. No statistically significant differences were found for exclusions, while regarding the completion of questionnaires, a higher proportion of completion was found for respondents who first answered the MACBETH protocol.

The number of exclusions associated both with MACBETH and the TTO variant, only due to MACBETH, and only due to the TTO variant were 33, 98 and 27, respectively. The main reasons for exclusions associated with MACBETH were: logical inconsistencies in the MACBETH matrix (43.6%) and logical inconsistency resulting from the rank order (8.6%). For the TTO variant, the main exclusions were for states better and worse than death given a zero score, 7% and 20.2%, respectively; and 51.9% and 4.1% of respondents showed logical and serious logical inconsistencies, respectively.

A valid sample for analysis with 210 individuals was obtained: 112 (46%) valid judgments associated with MACBETH and 183 (75%) valid judgments associated with the TTO variant; only 85 (40.5%) individuals had valid responses for both the TTO variant and MACBETH (note that at this stage different consistency criteria for exclusion have been applied for each method, with more demanding criteria being used for MACBETH, as will be later discussed).

### Sample

Table 2 shows the main characteristics of the study sample. The sample includes: a slight majority of women (63.8%); age range from 18 to 74 with mean age of 34 years old (SD=13); the majority of respondents being single; and almost 73.8% of respondents with a high educational level. All respondents expressed their own health states, having mostly placed themselves in very good health states, with 21.4% and 24.3% reporting moderate problems of pain/discomfort and anxiety/depression, respectively.

**Table 2:** Study sample characteristics

		Sample of respondents who completed the questionnaire (*n*=243)	Sample of respondents after exclusions due to MACBETH and TTO variant (*n*=210)
Gender (%)	Female	64.0	63.8
	Male	36.0	36.2
Age (%)	Mean (SD)	34.4 (13.1) years	34.3 (13.1) years
	18-24 years	31.7	33.3
	25-30 years	18.9	17.6
	31-44 years	25.5	25.2
	> 44 years	23.9	23.8
Educational attainment (%)	Less than secondary	2.1	1.4
	Secondary	26.3	24.8
	High than secondary	71.6	73.8
Marital status (%)	Single	51.0	50.0
	Married/ living with a partner	45.7	48.1
	Divorced/separated	2.5	1.4
	Widowed	0.8	0.5
Occupational status (%)	Student	33.3	36.2
	Employed	53.9	51.4
	Unemployed	9.7	3.3
	Retired	4.1	4.3
	Domestic	1.2	1.4
	Other situation	3.7	3.3
Household (%)	1-2 members	28.4	26.7
	3-4 members	63.0	64.3
	5 or more members	8.4	9.0
Chronic disease (%)	Yes	19.3	18.6
	No	77.8	79.1
	Not answer/Didn’t know	2.9	2.4
Numeracy (%)	Mean (SD)	2.4 (0.8) right answers	2.4 (0.8) right answers
	0 right answers	2.9	2.9
	1 right answer	11.5	11.4
	2 right answers	27.6	26.2
	3 right answers	58.0	59.5

Differences between population subsamples were investigated, in particular between the total sample and the subsamples obtained after exclusions due to MACBETH and due to the TTO. No statistically significant differences at the 5% level were found.

### Health-state values

The number of valuations per health state, descriptive statistics for the evaluations of hypothetical health states, the percentage of negative valuations, and the difference between mean values are reported in Table 3. The mean health-state value-score for MACBETH is 0.42 (SD=0.29) with a range between -0.080 (33333) and 0.860 (12111); for the TTO variant the mean value-score is 0.35 (SD=0.37), with a range between -0.446 (33333) and 0.831 (11121) being observed. For MACBETH the only health state with a negative average value was 33333, with more states with average negative values being observed for the TTO variant. Despite substantial differences between some health states, mean values do not differ remarkably: the absolute difference is greater than 0.1 for 9 health states (36%) and greater than 0.05 for 15 health states (60%).

**Table 3:** MACBETH and TTO evaluations for the 25 hypothetical EQ-5D-3L health states

State	MACBETH			TTO variant			MACBETH-TTO	Official TTO study^a^ (observed mean)	Official TTO study^b^ (observed mean)
	N	Mean ± SE	% neg.	n	Mean ± SE	% neg.	Dif.	Mean	Mean
11112	26	0.85 ± 0.03	0	48	0.81 ± 0.03	0	0.040	0.757	0.784
11113	31	0.58 ± 0.07	10.8	42	0.56 ± 0.06	7.1	0.012	0.344	0.412
11121	27	0.85 ± 0.02	0	47	0.83 ± 0.03	0	0.023	0.766	0.770
11131	28	0.65 ± 0.04	0	46	0.56 ± 0.05	2.2	0.095	0.283	0.319
11133	26	0.34 ± 0.07	15.4	48	0.40 ± 0.07	8.3	-0.062	0.112	0.186
11211	31	0.84 ± 0.02	0	42	0.80 ± 0.03	0	0.038	0.696	0.710
11312	27	0.55 ± 0.03	0	47	0.53 ± 0.06	6.4	0.014	0.480	0.535
12111	28	0.86 ± 0.02	0	46	0.83 ± 0.02	0	0.032	0.676	0.669
13311	26	0.43 ± 0.05	7.7	48	0.50 ± 0.04	2.1	-0.065	-0.124	-0.020
13332	31	0.17 ± 0.06	29.0	42	0.01 ± 0.07	26.2	0.161	-0.111	-0.005
21111	27	0.81 ± 0.02	0	47	0.78 ± 0.03	0	0.033	0.702	0.681
21323	28	0.35 ± 0.04	3.6	46	0.36 ± 0.05	2.2	-0.007	0.124	0.094
22121	31	0.58 ± 0.05	3.2	42	0.68 ± 0.03	0	-0.095	0.416	0.527
22122	27	0.51 ± 0.04	0	47	0.59 ± 0.04	0	-0.086	0.425	0.462
22222	26	0.58 ± 0.04	0	48	0.46 ± 0.05	4.2	0.119	0.264	0.329
22233	28	0.26 ± 0.05	14.3	46	0.16 ± 0.07	17.4	0.101	-0.045	-0.021
23232	27	0.28 ± 0.04	11.1	47	0.01 ± 0.07	25.5	0.265	0.112	0.223
23313	28	0.29 ± 0.04	3.6	46	0.18 ± 0.06	13.0	0.114	-0.096	-0.100
32211	28	0.40 ± 0.05	3.6	46	0.26 ± 0.05	8.7	0.134	0.066	0.122
32223	26	0.11 ± 0.06	34.6	48	0.06 ± 0.07	16.7	0.045	-0.271	-0.098
32313	31	0.14 ± 0.06	32.3	42	0.14 ± 0.08	21.4	0.011	-0.141	0.010
33232	31	0.04 ± 0.06	32.3	42	-0.21 ± 0.07	50.0	0.253	-0.301	-0.174
33321	26	0.15 ± 0.06	26.9	48	0.07 ± 0.08	20.8	0.073	-0.344	-0.217
33323	27	0.05 ± 0.04	33.3	47	-0.27 ± 0.08	51.1	0.315	-0.258	-0.127
33333	112	-0.08 ± 0.02	48.2	183	-0.45 ± 0.04	63.9	0.366	-0.497	-0.397
MAD: 0.1015									

^a^Overall sample from the Portuguese EQ-5D-3L valuation study [56].

^b^Respondents aged 18 to 49 years old from the Portuguese EQ-5D-3L valuation study [56].

Legend: *SE* Standard error, *MAD* Mean Absolute Difference

Table 3 also presents mean values for the same health states reported in the EQ-5D-3L Portuguese valuation study [56]. Here, the mean health-state value-score is 0.154 (SD=0.378) ranging between -0.497 (33333) and 0.766 (11121). From the health states valued, 10 were assigned negative values. Pearson’s correlation coefficients between the TTO variant and the Portuguese TTO tariff were found to be 0.92, and 0.96 between MACBETH and the Portuguese TTO tariff.

Given the particular characteristics of the sample used in this study – a younger and more educated sample than the population from [56] – we decided to compare results with a sub-sample of respondents from [56] aged between 18 and 49 years old. These assessments are reported in Table 3, showing a mean value of 0.227 (SD = 0.346), with negative values assigned to 9 health states. Values ranged from -0.397 (33333) to 0.784 (11112).

**Fig. 4:**
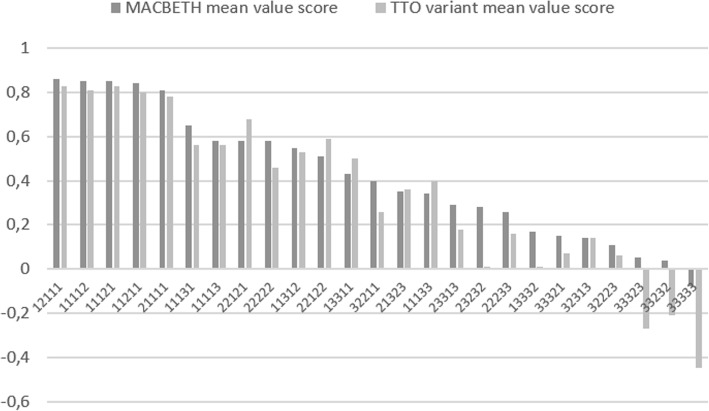
Comparison of mean scores obtained with MACBETH and with TTO value per health state, for a set of 25 health states

A comparison of mean scores obtained with MACBETH and with the TTO variant for the set of 25 health states ordered by MACBETH values is shown in Fig. 4. The x-axis in Fig. 4 depicts EQ-5D-3L health states ordered by decreasing order of MACBETH evaluations. It can be read that health state 12111 – depicting a health state with moderate problems regarding self-care and no problems on the other dimensions – corresponds to a value score of 0.86 (as compared to a score of 1 and 0 for full health and death, respectively). Pearson correlation coefficients for the evaluations obtained with both methods were determined: a correlation of *r* = 0.962 between the two mean scales, and a determination coefficient of *r*^2^ = 0.926, indicating that approximately 93% of the variability in one scale can be explained by the other.

In the analysis of whether each of the respondents’ numerical TTO evaluations are compatible with their own MACBETH qualitative judgments (taking Group 1 health states from Table 1), it was observed that all answers were incompatible.

### Preferences across elicitation protocols

Among the valid sample (210 individuals), 100 (47.6%) preferred expressing qualitative value judgments in words, associated with the MACBETH protocol, 46 (21.9%) preferred expressing numerical judgments with TTO, while 64 (30.5%) did not express a preference for one of those protocols.

Regarding the comments on the preferred elicitation protocol (total of 74), on the one hand 13 respondents considered the MACBETH task easier because it allows for comparing different health states side by side, and 17 found it not easy to provide numerical judgments with the TTO. On the other hand, 6 individuals preferred the TTO and considered it an easier elicitation protocol, and 6 individuals found the TTO to be less subject to respondents’ interpretation.

### Influence of numeracy

Regarding the numeracy evaluation, 59.5% of the respondents from the valid sample answered the 3 numeracy questions correctly, 26.2% answered 2 questions correctly, 11.4% answered only one question correctly, and 2.9% failed all questions.

**A. On the exclusion of answers**

No statistically significant differences (at a 5% level) were found between total exclusions of respondents with low and high levels of numeracy according to the short numeracy test. Nevertheless, we observed that the low numeracy sample (0-1 right numeracy answers) had a higher proportion of logical inconsistencies for the TTO variant than the high numeracy one (60% vs. 40%).

**B. On health-state evaluations**

Few differences on health-state evaluations were obtained for both protocols for low numeracy (0-1 right answers) and high numeracy (2-3 right answers) respondents. The Mean Average Deviation for the TTO variant and for MACBETH are 0.120 and 0.128, respectively. Individuals with higher numeracy levels showed higher correlation coefficients between MACBETH and TTO variant scales, although these correlation coefficients have high levels for the whole sample (with the exception of the subsample with very low numeracy – “0 right answers” – that had a very small number of individuals and a 0.378 Spearman correlation, respondents with at least one correct numeracy question had Spearman correlations above 0.895).

**C. On preferences**

Individuals with lower numeracy (0-1 right answers) were mostly indifferent regarding the two protocols, while individuals with higher numeracy (2-3 right answers) mostly preferred MACBETH. To determine the influence of numeracy level on individuals’ preferences we performed a multinomial logistic regression, with the dependent variable “preference” (categorical variable with three levels: MACBETH; TTO; and indifferent) and the independent variable “numeracy level” (numerical variable that varies between 0 and 3). Using the indifferent category as the baseline, numeracy was shown to have a statistically significant impact on the preference for MACBETH in comparison to the reference level (OR = 2.50 (95% CI: 1.63-3.86)), suggesting that individuals with higher numeracy levels are more likely to prefer expressing their judgments qualitatively than being indifferent. No statistically significant distinction was found between TTO and the indifferent level (OR = 1.30 (95% CI: 0.87-2.14)). When one considers the TTO category as a reference, higher numeracy was again found to be statistically associated with a preference for the use of the MACBETH protocol (OR = 1.83 (95% CI: 1.13-3.00)).

## Discussion

This article reports on a pilot study that has developed and tested a novel non-numerical protocol for valuing health states based on the MACBETH approach. An experimental design was built so as to analyse evaluations with that protocol, as well as differences and preferences of evaluators in answering to the non-numerical and numerical protocols. Results suggest that there is space to develop non-numerical protocols for health-state elicitation.

Discussion of results should acknowledge that, as expected, the use of a web survey was associated with a younger and more educated set of respondents than the average Portuguese or the population sample obtained in other evaluation studies, such as in [56] (our sample, however, is representative of the Portuguese population in terms of gender and marital status). The fact that our sample has two-thirds with high than secondary education should be interpreted as a sample bias. While internet experiments have emerged as a way of obtaining large representative data sets with relatively low costs [13], the use of web surveys has been reported to be challenging for eliciting TTO tariffs and other elicitation techniques [66] and potentially entails specific features and biases’ that may be affected by the questioning mode [67].

Another limitation of this study is that the adopted TTO protocol is a variant of the original TTO protocol, which was selected because of its implementation within a web survey format: respondents were asked to directly indicate the indifference point between alternatives whereas in the original TTO protocol a ping-pong approach is used to implement a choice-based iteration process to obtain an indifference value. However, this simplification may bear a closer conceptual resemblance to a visual analogue scale than to a TTO. These aspects should be considered when interpreting the results. To minimize this limitation, we have also compared the results with those of the EQ-5D-3L Portuguese valuation study where the regular TTO was used, and we found substantial changes that may be explained by differences in the sample characteristics.

Through the comparison of the MACBETH and TTO variant protocols, we concluded that a large number of inconsistencies in judgments was observed for both protocols. Comparison of excluded observations in MACBETH and the TTO variant should acknowledge that different exclusion criteria are applied. In the process of selecting observations in our sample, the total number of exclusions was higher for MACBETH mainly due to the application of more demanding consistency criteria, namely: an inconsistent MACBETH matrix of judgments obtained (43.6%) and logical inconsistency resulting from the rank order exercise (8.6%). If we had applied to the TTO variant the exclusions due to logical (including serious) inconsistencies analogous to ones applied to MACBETH, then a larger number of exclusions would apply to the TTO – 51.9% and 4.1% for logical and serious logical inconsistencies, respectively. These results suggest that it is important to develop methods that are designed to minimize inconsistent judgments and/or request that respondents address their inconsistencies during the elicitation process and correct their inconsistent judgments.

Analysis of the dropouts from the web survey shows a lower dropout for respondents facing the qualitative protocol first, which may suggest an ease of understanding of the MACBETH protocol. This conclusion is reached because of the higher proportion of respondents preferring that protocol and by the analysis of respondents’ comments.

In relation to health-state evaluations generated by the two protocols, in general mean value scales were found not to be dissimilar; however, values obtained with MACBETH were found to be higher, in particular for more severe health states. For health state 33333, the value obtained by the TTO variant is -0.446 and for MACBETH -0.080. This difference may partly be explained by the rescaling of negative values through the monotonic transformation, a procedure that we have explained as unnecessary when an interval scale is adopted. It is also worth noting that MACBETH and TTO variant results were also compared with data that came from the EQ-5D-3L valuation study [56], collected with the regular TTO, with differences being observed. These differences may be explained not only by the sample age and education biases, but also by the rescaling of negative values. Despite a high correlation between the (mean) value scales produced with the MACBETH and the TTO variant protocols, when the compatibility of MACBETH qualitative judgments with the TTO variant for each respondent was analysed, inconsistencies between qualitative and quantitative judgments were found for all group 1 respondents. This can be explained by the fact that participants provided qualitative judgments while not having the opportunity to discuss or reflect or revise their qualitative judgments, as is common when the MACBETH constructive approach is fully applied in face-to-face settings; and similarly, the adopted TTO variant did not implement the choice-based iteration process underlying the conventional TTO. It would be relevant to explore in future research additional procedures so that participants can reflect upon the consequences of their judgments and eventually adjust the produced numerical (health-state) scales.

When comparing our value scores with the mean value scales obtained with the Portuguese TTO tariffs for EQ-5D-3L reported in [56], we observed a higher level of correlation with non-numerical evaluations than with the TTO variant evaluations. Reasons that may explain this result are: we used a simplified adjustment of the commonly used protocol of the TTO; we used a web survey format that did not ask respondents to iterate, revise or validate their answers or offer facilitators’ help to respondents; and our sample of respondents was younger and more educated than the sample of respondents from [56].

Regarding the influence of the numeracy level on respondents’ evaluation and on the preference for distinct protocols, we observe that, in general, increasing numeracy seems to be associated with a higher preference for the non-numerical protocol. This result contradicts findings from previous preference elicitation studies [26, 27] that showed that respondents with higher numeracy preferred expressing values in numbers. Although there are few behavioural studies in this area, and existing studies have been developed in distinct contexts, one should analyse results in light of the characteristics of the adopted short numeracy test and of differences between protocols. Firstly, the adopted numeracy test has been most commonly used in decision-making under uncertainty contexts and captures probabilistic reasoning and statistical numeracy [68]. Nevertheless, as the MACBETH and TTO variant protocols make respondents answer in contexts of riskless choices (e.g. the methods in use do not make explicit considerations of uncertainty [27]), the results should be cross-checked with those of other numeracy tests for non-risk settings. Hence, future research may explore other numeracy tests that do not test probabilistic reasoning per se but, rather, the respondent's ability to deal with numbers as discussed and tested by [27]. Secondly, analyses of results should acknowledge that in web settings, respondents may make use of calculators to provide answers, and one is not capturing true numeracy [69]. Finally, the MACBETH and the TTO variant protocols further differ in terms of adopting numerical and non-numerical questions, and they differ by using difference-based and trade-off-based questioning protocols as well. Hence, our results may also be explained by other key features associated with both questioning protocols. Furthermore, regarding the testing of numeracy, it is also relevant in future studies to explore the effect of fluency on preference, as in [27].

Several of the limitations pointed out in our study can be overcome with future research. Firstly, methods in which respondents can revise their judgments and validate the health-state scales in line with their judgments can be devised. Specifically, the elicitation of health-state values can be further developed so that the conventional TTO protocol is used with extra consistency procedures, and the use of MACBETH is extended so that individuals adjust and validate a numerical scale after providing qualitative judgments (within a constructive process). Secondly, the use of a random and representative sample of respondents, as opposed to the non-probabilistic sampling method that we used for exploratory purposes (specifically the snowball sampling method), should be combined with non-numerical protocols. Thirdly, data could be collected by personal interviews and with the help of facilitators, therefore potentially obviating the limitations associated with web surveys. Fourthly, research should explore whether respondents in distinct contexts may be allowed to choose a specific questioning protocol. Finally, research could explore the use of distinct numeracy tests devised for contexts of riskless choices and use mechanisms to better understand respondents’ choices.

## Conclusions

In this study, we propose an innovative and unconventional approach to evaluate health states, based on the use of the MACBETH (qualitative) questioning mode to value EQ-5D-3L health states and conduct a behavioural experiment to examine the extent to which respondents’ numeracy impacts their preferences for two different preference elicitation techniques for health-state evaluation, one numerical and one non-numerical. We explore these two techniques as, according to the literature, they might not be psychologically equivalent (i.e., not being perceived and experienced in the same manner by individuals) and they may eventually address cognitive issues reported in the health-state preference elicitation literature.

Results suggest that it is worth considering the use of non-numerical preference elicitation methods in health, highlighting the fact that obtained values are consistent, and individuals have shown a higher preference for this mode of expressing value judgments. Non-numerical protocols may be seen as less cognitively demanding and MACBETH provides a simplified and unique protocol for states better and worse than death. Our results further show that health-state elicitation ought to consider the respondents’ preference for protocols, and also highlight the distinctive advantages of numerical and non-numerical techniques; in addition, it is worth noting that neither technique was absolutely preferred.

Several research paths have been identified, including exploring how to improve consistency in respondents’ assessments, replicating the study for a controlled population, improving web platforms for preference elicitation, looking into numeracy issues, and understanding better why individuals prefer one protocol. This study offers several insights for health research: it shows that respondents may prefer distinct protocols for questioning that should be researched to avoid cognitive difficulties and to enhance evaluations; there is a need to devise robust procedures so as to avoid evaluations depending on methods in use (as scores can, for instance, change health technology assessment results); more research should be devoted to the measurement of numeracy for health-preference elicitation contexts; and more behavioural research is required regarding preference-elicitation methodological choices and the implications of using distinct protocols for questioning.

## Abbreviations

EQ-5D-3L: EuroQol-5D
MACBETH: Measuring Attractiveness by a Categorical Based Evaluation Technique
QALY: Quality Adjusted Life Year
SF-6D: Short-Form 6D
TTO: Time Trade-Off
